# A Case Report of Urinary Bladder Carcinosarcoma and Review of the Literature

**DOI:** 10.1155/2011/415675

**Published:** 2011-06-30

**Authors:** Christos Zachariadis, Ioannis Efthimiou, Stylianos Giannakopoulos, Athanasios Bantis, Alexandra Giatromanolaki, Efthimios Sivridis, Stavros Touloupidis

**Affiliations:** ^1^Department of Urology, Democritus University of Thrace, Alexandroupolis, Alexandroupolis 68100, Greece; ^2^Department of Pathology, Democritus University of Thrace, Alexandroupolis, Alexandroupolis 68100, Greece

## Abstract

Carcinosarcoma of the bladder is an unusual tumour characterized by a combination of malignant epithelial and soft tissue elements. Most of the reported cases have been case reports or small series. Optimal treatment is uncertain. We herein report our experience in such a case treated with transurethral resection followed by radiotherapy with adverse final outcome. Treatment of bladder carcinosarcomas should be aggressive and multimodal but optional treatment is still unknown. Radiotherapy alone is insufficient as a treatment option of these aggressive tumors.

## 1. Introduction

Carcinosarcomas (CSs) of bladder cancer are rare tumors and consist of a combination of malignant epithelial and sarcomatoid components. They are more common in males than females with a ratio nearly 2 : 1. The most common age at presentation is the seventh decade of life [1]. Usual presentation is that of macroscopic hematuria and dysuria. Risk factors include previous pelvic radiation and cyclophosphamide therapy. Generally more than 70% of CSs present with advanced stage and have a worse prognosis than conventional urothelial carcinomas [1]. 

Hereby, we present a case of urinary bladder CS with mesenchymal elements of osteosarcoma and chondrosarcoma which received adjuvant radiotherapy after transurethral resection with no response, and we review the recent literature.

## 2. Case Presentation

**Figure 1:**
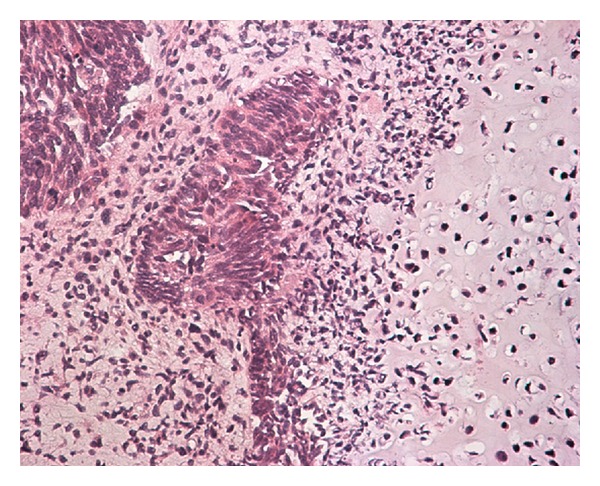
The epithelioid and sarcomatoid component of the tumor showing polygonal cells and spindle cells, respectively. There is focal appearance of osteosarcoma cells (hematoxylin-eosin, original magnification ×400).

A 76-year-old female with a past medical history of heart failure and non-insulin-depenent diabetes mellitus presented with a two-week history of total heamaturia and dysuria. Physical examination was unremarkable. Abdominal ultrasound revealed a mass located on the right lateral wall of the urinary bladder. Rigid cystoscopy revealed a 4-5 cm hemorrhagic, exophytic, and invasive lesion occupying the right lateral bladder wall and ureteral orifice. Computerized tomography (CT) of the abdomen showed a soft tissue mass with multiple calcified foci in the right bladder wall, extending posterior with dilation of the ipsilateral ureter. CT of the chest and bone scan was negative for metastases. The clinical stage was cT3N0M0. The patient underwent transurethral resection of the tumor. Histopathology of the specimen revealed bladder CS invading the bladder muscle. The epithelial component was high-grade urothelial cancer, and the sarcomatoid element consisted of osteosarcoma and chondrosarcoma (Figure 1). The patient made an uneventful recovery. Radical cystectomy was deemed too high risk due to severe heart failure of the patient and the local extension of the disease. The patient was treated with 50 Gy of 3D conformal radiotherapy but unfortunately did not complete the protocol due to progression of the disease. She developed renal failure and died five months later.

## 3. Discussion

The histological features of CS of the bladder vary. Macroscopically these tumors are usually large, polypoid or nodular. Most reported cases contain high-grade papillary/undifferentiated urothelial carcinoma although other subtypes like small-cell carcinoma, squamous carcinoma, and adenocarcinoma have been reported. The most common sarcomatous elements are chondrosarcoma, leiomyosarcoma, and malignant fibrous histiocytoma. In our case the sarcomatous element was a mixture of chondrosarcoma and osteosarcoma. 

There is considerable confusion and disagreement in the literature regarding nomenclature. In some series, both CS and sarcomatoid carcinoma (SC) are included as “sarcomatoid carcinoma” and these terms are used interchangeably [2]. In others they are regarded as separate entities [1]. An interest study by Wright et al. showed a different survival rate between patients with CS and SC (5-year survival rate 17% versus 37%), offering some justification for the continued differentiation of these tumor types for clinical prognostication [1].

Differential diagnosis should also be considered with urothelial carcinomas with osseous metaplasia, primary osteogenic sarcoma, especially if the sarcomatous elements in the former condition produce osteoid and nonneoplastic conditions like encrusted cystitis and polypoid cystitis glandularis. 

 CS carries a dismal prognosis with a median cancer-specific survival of 14 months (95% CI 7–21 months) [3]. Many series emphasize the importance of stage for the prognosis [1, 3, 4]. Half of patients die within 1 year from the diagnosis. However, in a case series study none of 8 patients with pT1 disease died of the disease, although followup was incomplete in 3 of them [4]. 

Due to rarity of the tumor, all the treatment results are from case reports and small case series [2, 5]. A variety of treatment modalities have been described but optimal treatment requires rather a multimodality therapy (Table 1). Transurethral resection and partial cystectomy carry the risk of incomplete tumor resection. Radical cystectomy with pelvic lymphadenectomy is the mainstay of treatment [1], although patients tend to develop local recurrence after surgery [1, 3]. In a recent study which analyzed retrospectively 221 cases the overall 5-year cancer-specific survival rate after cystectomy was only 20.3%, suggesting a high risk of early dissemination [3]. Cancer-specific survival was significantly better for those who underwent cystectomy instead of transurethral resection [3].

 Adjuvant radiotherapy and various combinations of chemotherapy have yielded inconsistent results. In the study by Wright et al. a small cohort of 9 patients received radiation after transurethral resection but subgroup survival analysis was not available. Radiotherapy alone is rather insufficient as it was shown in our case. In our opinion poor treatment results are also due to either the advanced stage of the disease or the poor status of the patients selected for radiotherapy as they are not candidates for more aggressive treatment. Carboplatin and gemcitabine have been used in combination with radiotherapy with promising results considering that traditional standard treatment for most sarcomas show poor response to primary radiation therapy [6–9].

## 4. Conclusion

Treatment of bladder carcinosarcomas should be aggressive and multimodal but optional treatment is still unknown. Radiotherapy alone is insufficient as a treatment option of these aggressive tumors.

## Figures and Tables

**Table 1:** Treatment modalities for the bladder carcinosarcoma.

Transurethral resection
Transurethral resection + radiation
Transurethral resection + radiation + chemotherapy

Cystectomy
Cystectomy + radiation
Cystectomy + radiation + chemotherapy
Partial cystectomy
